# Identification of Early Biochemical Recurrence Predictors in High-Risk Prostate Cancer Patients Treated with Carbon-Ion Radiotherapy and Androgen Deprivation Therapy

**DOI:** 10.3390/curroncol30100636

**Published:** 2023-09-27

**Authors:** Takanobu Utsumi, Hiroyoshi Suzuki, Hitoshi Ishikawa, Masaru Wakatsuki, Noriyuki Okonogi, Masaoki Harada, Tomohiko Ichikawa, Koichiro Akakura, Yoshitaka Murakami, Hiroshi Tsuji, Shigeru Yamada

**Affiliations:** 1 Department of Urology, Toho University Sakura Medical Center, 564-1 Shimoshizu, Sakura-shi, Chiba 285-8741, Japan; takanobu.utsumi@med.toho-u.ac.jp (T.U.); hiroyoshi.suzuki@med.toho-u.ac.jp (H.S.); 2 QST Hospital, National Institutes for Quantum Science and Technology, 4-9-1 Anagawa, Inage-ku, Chiba-shi, Chiba 263-8555, Japan; wakatsuki.masaru@qst.go.jp (M.W.); okonogi.noriyuki@qst.go.jp (N.O.); m-harada@joy.ocn.ne.jp (M.H.); tsuji.hiroshi@qst.go.jp (H.T.); yamada.shigeru@qst.go.jp (S.Y.); 3 Department of Urology, Chiba University Graduate School of Medicine, 1-8-1 Inohana, Chuo-ku, Chiba-shi, Chiba 260-8670, Japan; tomohiko_ichikawa@faculty.chiba-u.jp; 4 Department of Urology, Japan Community Health-Care Organization Tokyo Shinjuku Medical Center, 5-1 Tsukudo-cho, Shinjuku-ku, Tokyo 162-8543, Japan; akakurak@ae.auone-net.jp; 5 Department of Medical Statistics, Faculty of Medicine, Toho University, 5-21-16 Omori-nishi, Ota-ku, Tokyo 143-8540, Japan; yoshitaka.murakami@med.toho-u.ac.jp

**Keywords:** biochemical recurrence, early recurrence, prostate cancer, high-risk, carbon-ion radiotherapy, androgen deprivation therapy

## Abstract

The aim of this retrospective study was to identify clinical predictors of early biochemical recurrence (BCR) in patients with high-risk prostate cancer (PCa) treated with carbon-ion radiotherapy (CIRT) and androgen deprivation therapy (ADT). A total of 670 high-risk PCa patients treated with CIRT and ADT were included in the study. Early BCR was defined as recurrence occurring during adjuvant ADT after CIRT or within 2 years after completion of ADT. Univariate and multivariate analyses were performed to identify clinical predictors of early BCR. Patients were also classified according to the Systemic Therapy in Advancing or Metastatic Prostate cancer (STAMPEDE) PCa classification. Early BCR was observed in 5.4% of the patients. Multivariate analysis identified clinical T3b stage and ≥75% positive biopsy cores as clinical predictors of early BCR after CIRT and ADT. The STAMPEDE PCa classification was also significantly associated with early BCR based on univariate analysis. These predictors can help clinicians identify patients who are at risk of early BCR. In the future, combination therapy of ADT with abiraterone may be an option for high-risk PCa patients who are at risk of early BCR, based on the results of the STAMPEDE study.

## 1. Introduction

Prostate cancer (PCa) ranks as the second most prevalent malignancy and the primary cause of cancer-related death in the male population worldwide [1,2]. The incidence and mortality rates of PCa demonstrate an upward trajectory due to age-associated trends of oncogenesis in numerous countries, despite advancements in diagnostic and therapeutic modalities [3,4]. PCa severity exhibits heterogeneity, ranging from indolent to highly aggressive phenotypes, thus complicating prognostic predictions and determination of patient-specific treatment approaches. Consequently, PCa patients are typically categorized into risk strata predicated on the prostate-specific antigen (PSA) concentration, clinical T classification, and biopsy-derived Gleason score (GS) [5,6]. High-risk PCa constitutes 20–30% of PCa diagnoses [7,8].

Both radiotherapy (RT) and radical prostatectomy (RP) play crucial roles in the definitive management of non-metastatic localized or locally advanced PCa [9]. Despite the development of conventional external beam RT, over 50% of high-risk PCa patients will exhibit biochemical recurrence (BCR) over long-term follow-up, correlating with metastases and cancer-specific survival (CSS) [10]. At present, there is no unanimous consensus regarding the optimal treatment recommendations for high-risk PCa patients in clinical guidelines [6,11].

Carbon-ion radiotherapy (CIRT) for localized PCa was initiated in 1995 by the National Institute for Quantum Science and Technology in Chiba, Japan (previously named the National Institute of Radiological Science) [12]. The physical and biological characteristics of CIRT result in superior effects on neoplastic cells and an optimal dose distribution [13]. Favorable clinical outcomes, including reduced BCR and CSM rates, have been observed in high-risk PCa patients undergoing CIRT and androgen deprivation therapy (ADT) [14]. Moreover, CIRT in conjunction with long-term ADT has produced superior treatment outcomes in high-risk PCa patients compared with RT and RP treatments [13,14]. Nonetheless, some high-risk PCa patients treated with CIRT and ADT exhibit an elevated BCR risk, which is a clinical concern because of the association between BCR and overall mortality [13,14,15].

Early BCR occurring after definitive treatment exhibits a strong correlation with unfavorable prognoses in PCa patients [16,17,18]. Patients with PCa, regardless of risk classification, who experienced early BCR following primary RT or RP exhibited significantly elevated risks of distant metastases and PCa mortality [16,17,18]. These findings underscore the importance of identifying high-risk PCa patients at risk of early BCR to facilitate precise and effective treatments, thereby enhancing survival outcomes. Additionally, recent investigations have postulated that early BCR may be correlated with the presence of micro-metastases at the time of diagnosis or the emergence of aggressive tumor features during disease progression [19,20]. Consequently, early BCR may be a characteristic of high-risk PCa necessitating aggressive and tailored treatment strategies. Given the lack of studies evaluating the patterns and predictors of early BCR in high-risk PCa patients treated with CIRT and ADT, identifying clinical predictors of early BCR in this population will enable clinicians to refine patient selection, treatment planning, and personalized follow-up strategies.

More aggressive concomitant ADT is a treatment option for patients at high risk of early BCR. Recently, Attard and colleagues disseminated a meta-analytical exposition of principal findings stemming from two randomized, controlled Phase III trials within the Systemic Therapy in Advancing or Metastatic Prostate Cancer (STAMPEDE) platform protocol [21]. The investigation encompassed individuals afflicted by high-risk, non-metastatic prostate carcinoma, including those presenting with high-risk, non-metastatic PCa (N0 or N1), who underwent androgen deprivation therapy (ADT) and radiotherapy (RT) for a duration of 3 years. The treatments arms were ADT alone versus ADT with 2 years of abiraterone and prednisolone. The results of the study showed that ADT with abiraterone and prednisolone was superior to ADT alone in terms of several prognostic outcomes. Consequently, the authors advocated for RT combined with ADT and 2 years of abiraterone and prednisolone as the new gold standard for high-risk non-metastatic PCa [21]. The STAMPEDE risk classification diverges from conventional risk stratification systems, such as D’Amico and National Comprehensive Cancer Network (NCCN). High-risk PCa according to STAMPEDE is defined by at least two of the following criteria: PSA level ≥ 40 ng/mL, clinical (cT) 3 or 4 stage, or GS8–10. However, no existing studies on PCa patients treated with CIRT and ADT have validated the STAMPEDE risk criteria.

The objective of this investigation is to reclassify patients afflicted by high-risk PCa who have undergone treatment with CIRT and ADT utilizing the STAMPEDE risk criteria and to identify clinical predictors of early BCR following CIRT and ADT. The findings of this study may offer invaluable insights into the risk stratification of this patient population, potentially enhancing treatment outcomes for this patient subset.

## 2. Materials and Methods

### 2.1. Patients

This retrospective analysis scrutinized data derived from prospective clinical trials conducted at the Research Center for Charged Particle Therapy, QST Hospital. The study was approved from the institutional review board at QST Hospital (Approval No. N21-005), and potential participants were given the opportunity to opt-out of enrollment. The study enrolled patients diagnosed with high-risk PCa who underwent CIRT in combination with ADT between April 2000 and May 2018. The inclusion criteria encompassed patients with pathologically verified PCa, staged as T1–T3N0M0, devoid of concomitant primary malignancies and void of any precedent interventions for PCa. In accordance with the TNM Classification of Malignant Tumors, TNM staging was ascertained through digital rectal examination, ultrasonography, computed tomography (CT), magnetic resonance imaging (MRI), and isotope bone scintigraphy [22]. High-risk PCa is delineated at QST Hospital as an initial PSA concentration of ≥20 ng/mL, clinical stage T3a/b ailment, and/or GS ≥ 8, aligning with the categories of high-risk and very high-risk disease (excluding stage cT4) in accordance with the National Comprehensive Cancer Network (NCCN) classification [6]. Each prostate biopsy specimen underwent reassessment under the purview of a solitary pathologist (M.H.) to determine the percentage of positive biopsy cores (%PC) and the Gleason score. Of 929 patients, 670 (72.1%) had sufficient data and were therefore included in this study.

### 2.2. CIRT

Patients received CIRT at QST Hospital, Japan, according to a comprehensive treatment protocol reported previously [12,13,14,15]. CIRT targeting the prostate gland and seminal vesicle was administered once a day for 4 days per week. The radiation dose was measured in Gy (RBE) (physical carbon ion dose [Gy] × RBE). The RBE value for CIRT was estimated as 3.0 at the distal portion of the spread-out Bragg peak in prior investigations [12,13,14,15]. The following dose fractionation scheme was employed in this study: 63.0 Gy (RBE) administered over 20 fractions (77 cases), 57.6 Gy (RBE) administered over 16 fractions (298 cases), and 51.6 Gy (RBE) administered over 12 fractions (295 cases).

### 2.3. ADT

All patients underwent neoadjuvant and concomitant ADT for 2–6 months prior to CIRT. A luteinizing hormone-releasing hormone analogue was used for ADT and was subcutaneously injected with or without an oral anti-androgen, such as bicalutamide or flutamide. No patient underwent surgical castration in this study. Adjuvant ADT after CIRT was recommended for a minimum of 24 months, inclusive of the duration of neoadjuvant ADT. Patients treated with ADT for over 36 months total were excluded from the study due to a potential long-term effect of ADT on obscuring BCR.

### 2.4. Assessments

Patients were examined at 3-month intervals for the initial 5 years after CIRT and at 3- to 6-month intervals subsequently. The follow-up interval was defined as the period from CIRT initiation to the last follow-up visit. Clinical records were retrieved and updated in August 2022. BCR was defined as a PSA level ≥ 2.0 ng/mL above the nadir according to the Phoenix definition [23]. The primary endpoint of this study was early BCR, which was defined as BCR occurring during adjuvant ADT or within 2 years after ADT completion. BCR-free survival was calculated from the date of CIRT initiation. 

### 2.5. Statistical Analysis

First, clinicopathological characteristics were compared between early and non-early BCR. Continuous variables were juxtaposed through Student’s *t*-tests or the Mann–Whitney U-test, while categorical variables underwent comparison via the Chi-square test or Fisher’s exact test. Second, the following variables were analyzed as candidate predictors of early BCR: age, PSA level, cT stage, biopsy GS, and %PC. The cut-off value of each variable was the value associated with the maximum area under the survival curve. The associations between early BCR and the candidate predictors were assessed using Gray’s test, as univariate analysis, and Fine-Gray subdistribution hazard model, as multivariate analysis. As the STAMPEDE risk criteria overlap with these predictors, Gray’s test was conducted separately. Third, the association between early BCR and CSS was evaluated using Gray’s test. Mortality unrelated to prostate cancer was regarded as a competing risk in the analysis. *p* < 0.05 was defined as statistical significance. All statistical analyses were performed using SPSS Statistics 23 (IBM, Chicago, IL, USA) and EZR software (http://www.jichi.ac.jp/saitama-sct/SaitamaHP.files/statmed.html, accessed on 1 July 2023) [24].

## 3. Results

A total of 670 patients diagnosed with high-risk PCa were included in the analysis. Table 1 presents the following patient characteristics: age, PSA level, cT stage, GS, %PC, ADT duration, CIRT dose, early BCR rate, and time to early BCR. The patients had a mean age of 68.1 (standard deviation [SD], 6.9) years and median PSA level of 12.7 (interquartile range [IQR], 7.2–24.3) ng/mL. The cT stage was cT1 in 110 (16.4%) patients, cT2a in 132 (19.7%), cT2b in 91 (13.6%), cT2c in 37 (5.5%), cT3a in 239 (35.7%), and cT3b in 61 (9.1%). The GS was GS3+3 in 22 (3.3%) patients, GS3+4 in 93 (13.9%), GS4+3 in 103 (15.4%), GS4+4 in 135 (20.2%), GS3+5 in 25 (3.7%), GS4+5 in 255 (38.1%), GS5+4 in 34 (5.1%), and GS5+5 in 3 (0.4%). The median %PC was 41.7% (IQR, 25.0–60.0%). The median follow-up interval was 81.6 (IQR, 53.7–115.9) months, and the median duration of androgen deprivation therapy (ADT) was 25.0 (IQR, 24.0–27.0) months. Thirty-six patients (5.4%) experienced early BCR, and the time to early BCR was 42.8 (IQR, 29.0–45.3) months.

Table 2 presents the STAMPEDE risk stratification of the patients with high-risk PCa defined by conventional risk criteria. Based on the STAMPEDE criteria, 180 (26.8%) patients were classified as high risk. The STAMPEDE risk factors were PSA level ≥ 40 ng/mL (78, 11.6%), cT3 or higher stage (300, 44.8%), and GS ≥ 8 (452, 67.5%). Table 3 presents the clinicopathological characteristics of patients with early BCR and non-early BCR. Patients with early BCR exhibited a higher PSA level, cT stage, %PC, and rate of high-risk PCa defined by the STAMPEDE risk criteria.

Table 4 presents the results of the univariate and multivariate analyses of the associations between the clinical variables and early BCR. In the univariate analyses, cT3b stage, PSA level ≥ 50 ng/mL, and %PC ≥ 75% were significant predictors of early BCR. In the multivariate analysis, cT3b stage (hazard ratio [HR]: 3.63, 95% confidence interval [CI]: 1.76–7.49, *p* < 0.01) and %PC ≥ 75% (HR: 3.51, 95% CI: 1.84–6.69, *p* <0.01) were significant predictors of early BCR. When cT3b stage and %PC ≥ 75% were examined, the odds ratio was 2.1, which is not high enough to suspect multicolinearity. Therefore, in the multivariate analysis, cT3b stage and %PC ≥ 75% were included together.

**Figure 1:**
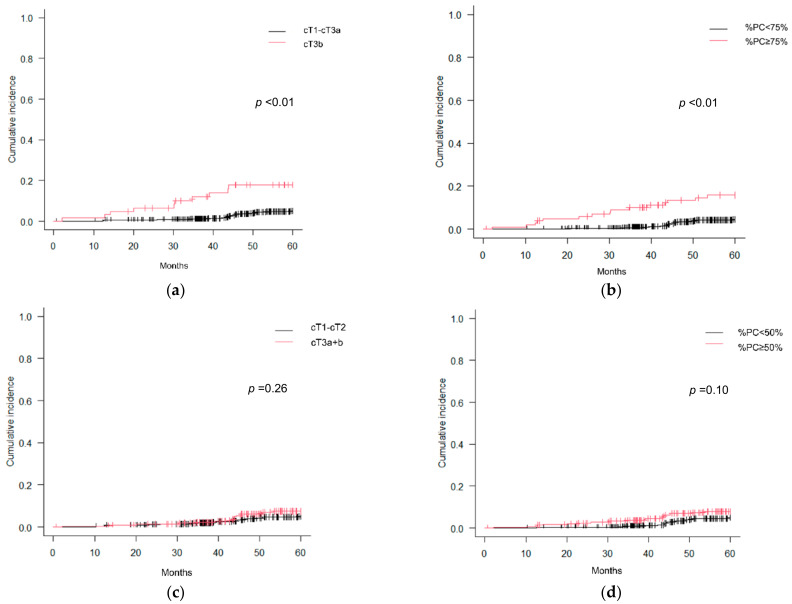
Cumulative incidence curves for early BCR: (**a**) cT3b; (**b**) %PC ≥ 75%; (**c**) cT3a+3b; (**d**) %PC ≥ 50%.

**Figure 2:**
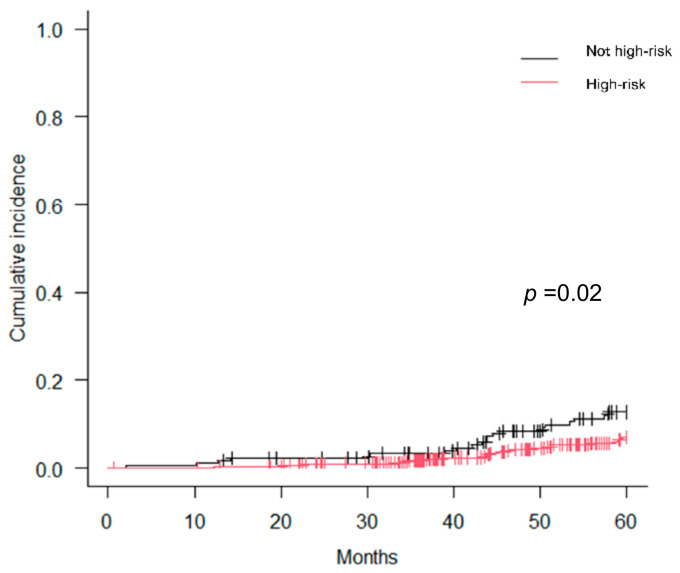
Cumulative incidence curves for early BCR according to STAMPEDE high-risk.

**Figure 3:**
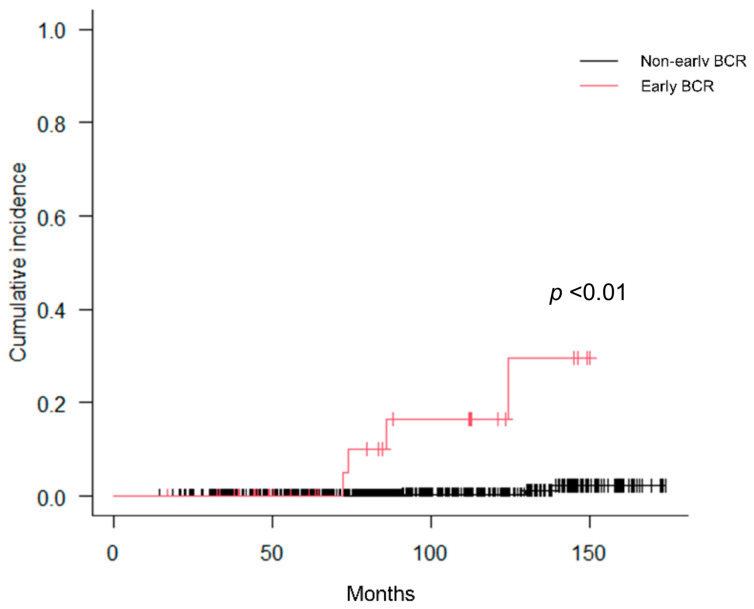
Cumulative incidence curves for CSS according to occurrence of early BCR.

Figure 1 presents the cumulative incidence curves pertaining to early BCR as stratified by cT stage and %PC. Notably, cT3b stage and %PC ≥ 75% demonstrated a significant association with early BCR, whereas cT3a+b stage and %PC ≥ 50% did not exhibit such a correlation. Figure 2 presents the cumulative incidence curves for early BCR based on the STAMPEDE risk classification. The STAMPEDE high-risk classification was significantly associated with early BCR. Figure 3 presents the cumulative incidence curves for CSS according to the occurrence of early BCR. Occurrence of early BCR was significantly associated with CSS.

## 4. Discussion

In this study, we assessed the outcomes of early BCR in a cohort of 670 patients afflicted by high-risk PCa who underwent CIRT in conjunction with ADT. The present study demonstrated a good outcome after CIRT combined with ADT in high-risk PCa patients, as the cumulative incidence of early BCR was only 5.4% in our cohort. The clinical advantages of CIRT over conventional RT for high-risk PCa, including higher local control rates and lower toxicity rates, have been reported in several studies [12,13,14,15]. Generally, PCa patients experiencing early BCR, compared with later BCR, after primary RT exhibit considerably increased risks of distant metastases and PCa mortality [17,18]. Our previous study indicated that BCR after CIRT and ADT is an independent prognostic factor for overall survival and CSS [14]. The present study also demonstrated that early BCR is significantly correlated with CSS (Figure 3). Hence, it is important to identify clinical factors that predict early BCR in high-risk PCa patients after CIRT and ADT.

Common predictors of BCR after RT, not necessarily those specific to early BCR, include PSA level ≥ 20 ng/mL, cT stage ≥ 3, and GS ≥ 8, according to the D’Amico and NCCN risk classification systems [5,6]. Furthermore, the PSA nadir within 12 months is an early surrogate marker of BCR after external beam RT or low-dose-rate brachytherapy for all-risk PCa [25,26]. Although not examined in the present study, PSA kinetics might also constitute a crucial prognostic factor for early BCR. The current study identified cT3b stage and %PC ≥ 75% as significant predictors of early BCR in the multivariate analysis (Table 4). cT3b stage and %PC ≥ 75% seem to reflect increased tumor invasion and volume although the actual extra-/intraprostatic GTV was not calculated using multiparametric magnetic resonance imaging (mpMRI) in each patient [27,28].

Utilizing advanced imaging modalities such as mpMRI and Positron Emission Tomography (PET) in the planning of RT for primary PCa necessitates specific prerequisites. These prerequisites encompass precise staging of both extraprostatic and intraprostatic tumor masses, robust delineation of the intraprostatic gross tumor volume (GTV), and a consistent characterization of the biological attributes of PCa [27,28]. Although our present study was conducted over a long period of time and the latest imaging modalities were not used in all patients, both mpMRI and prostate-specific membrane antigen PET (PSMA-PET)/CT excel in accurately detecting extraprostatic and intraprostatic tumor burdens, significantly influencing RT strategies [28]. On the other hand, certain studies have posited that mpMRI and PSMA-PET provide complementary information for intraprostatic GTV delineation [28]. Furthermore, variability among observers has been noted in intraprostatic tumor delineation relying on mpMRI [27]. Presently, it remains uncertain whether PET-based GTV delineation is also susceptible to interobserver heterogeneity [28]. Subsequent research including CIRT is warranted to elucidate whether multimodal imaging has the capacity to visualize biological processes inherent to prostate cancer pathophysiology, its resistance to radiation, and locally recurrence PCa after RT. Pre-radiation evaluation using multimodal imaging will be necessary, especially for high-risk PCa with robust local factors, such as cT3b and/or %PC ≥ 75%.

Roughly 90% of local recurrences manifest within the dominant lesions, denoting the most preeminent malignant lesion encompassed within the prostate [29]. Furthermore, in addition to imaging evaluation prior to RT, focal boost to extraprostatic and intraprostatic lesions needs to be validated in the future. Poon and colleagues systematically reviewed clinical outcomes for MRI-guided external beam RT (EBRT) focal boost to intraprostatic lesions [30]. MRI-guided EBRT focal boost for intraprostatic lesions in PCa was proven to be a feasible and safe approach, yielding low GI/GU toxicities and favorable biochemical disease outcomes. They concluded that level 1 evidence supports its superior BCR-free survival in comparison to whole-prostate irradiation using standard fractionation [30]. Clinical trials concerning focal boost therapy will be needed in the future for CIRT as well.

Our previously reported predictors of the 10-year BCR rate included age < 70 years, cT3a+b stage, GS ≥ 9, and %PC ≥ 50% [31], whereas predictors of early BCR in this investigation were cT3b stage and %PC ≥ 75%. We previously posited that the application of the Candiolo nomogram in high-risk PCa patients has the capability to forecast the 10-year BCR-free survival rate. However, in terms of early BCR, the Candiolo nomogram was not so useful due to heterogeneity in the risk level: 10 (27.8%) intermediate-risk patients, 13 (36.1%) high-risk patients, and 13 (36.1%) very high-risk patients out of 36 patients with early BCR. Therefore, clinicians should be especially cautious with high-risk PCa patients with cT3b and/or %PC ≥ 75%, resulting in early BCR after CIRT and ADT.

Next, we assessed tumor characteristics linked to an elevated susceptibility to early BCR in order to discern high-risk individuals who may potentially derive advantages from comprehensive systemic therapeutic approaches. Even subsequent to the administration of combined CIRT and ADT, individuals afflicted by high-risk PCa manifesting cT3b stage and/or a %PC ≥ 75% continued to exhibit a heightened vulnerability to early BCR. Therefore, novel aggressive therapeutic strategies are needed to improve the therapeutic outcomes of these patients. Various salvage therapies have been suggested for BCR after RT [32]. However, salvage therapy can only be used after BCR, and we prefer an adequate combination therapy to use before BCR.

In the present study, we investigated potential future indications for concomitant therapy. Recently, Attard et al. evaluated the efficacy of adding abiraterone and prednisolone with or without enzalutamide to ADT in patients with high-risk non-metastatic PCa [21]. The investigation involved 1974 patient datasets from the STAMPEDE multi-arm, multi-stage protocol, with the primary endpoint being metastasis-free survival. The results indicated that adding abiraterone and prednisolone significantly improved metastasis-free survival, overall survival, CSS, BCR-free survival, and progression-free survival compared with ADT alone, highlighting the advantages of combining RT with ADT and abiraterone for high-risk non-metastatic PCa [21]. Based on these findings, recent European Association of Urology guidelines recommend offering 2 years of abiraterone with ADT when administering definitive RT to non-metastatic high-risk PCa patients, including those with cN1 disease [33]. In the Advanced Prostate Cancer Consensus Conference in 2022, when asked about the preferred systemic therapy to add to local RT for high-risk PCa patients with N0M0 on next-generation imaging and GS8–10, 78% of panelists voted for 2–3 years of ADT with 2 years of abiraterone [34].

The most robust aspect of our study was investigating the STAMPEDE risk criteria to reclassify high-risk PCa. The original STAMPEDE criteria defined high-risk PCa as cN0 or cN1 with at least two of the following: PSA level ≥ 40 ng/mL, cT3 or T4 stage, and GS8–10. We found that 28.1% of our conventional high-risk PCa patients met the STAMPEDE high-risk criteria (Table 2), although patients with node positive or cT4 were not included. The STAMPEDE high-risk classification was significantly associated with the cumulative incidence of early BCR (Figure 3). This finding underscores the importance of using the STAMPEDE risk criteria to define high-risk PCa, as it ensures that the patient population can be matched to future systemic therapy. In particular, high-risk PCa patients with an increased risk of early BCR may benefit from 2 years of concomitant abiraterone therapy. Although it depends on the medical insurance system of each country, the addition of abiraterone to concomitant hormone therapy will be an option in the future. Thus, individualized therapeutic and follow-up strategies should be considered in the future due to distinct predictors of early versus late BCR. Recently, some researchers reported that MRI- or biology-guided focal boost therapies to intraprostatic lesions were feasible and safe, with low toxicities and favorable biochemical disease outcomes [30,35]. In the future, boost therapy with various modalities as well as the combined systemic treatments including abiraterone will be investigated in high-risk PCa patients who undergo CIRT.

This study has several limitations that should be considered when interpreting the results. First, as a retrospective study, it may have been subject to inherent biases that cannot be completely controlled for, despite the use of multivariate analysis to adjust for potential confounders. Second, this study included patients treated at a single institution only, which may limit the generalizability of the findings to other populations or treatment settings. Third, the sample size of the patients with early BCR was relatively small, which may have limited the statistical power to detect other potential predictors of early BCR. Fourth, intraprostatic GTV was not calculated using mp-MRI in this study, a part of the patients did not undergo mp-MRI. Also, our high-risk PCa patients did not undergo PSMA-PET/CT or choline PET/CT, so the possibility of micro-metastases in a few patients cannot be ruled out completely. The PCa patients at high risk for early BCR may benefit from PSMA-PET/CT to aggressively screen for micro-metastases in the future. Lastly, an effect of ethnicity on BCR cannot be ruled out, as this study was conducted mainly in Japanese patients.

## 5. Conclusions

The present study provides evidence that cT3b stage and %PC ≥ 75% are significant predictors of early BCR in patients with high-risk PCa treated with CIRT combined with ADT. These findings may have important implications for selecting patients who will benefit from this treatment approach and for developing future treatment strategies for high-risk PCa. Additionally, future clinical trials investigating the use of CIRT combined with ADT and other novel treatments, such as abiraterone, for high-risk PCa will be critical to determine the optimal treatment approach for these patients.

## Figures and Tables

**Table 1:** Patient characteristics.

Variable	*n* = 670
Age (years), mean (SD)	68.1 (6.9)
PSA level (ng/mL), median (IQR)	12.7 (7.2–24.3)
cT stage	cT1: 110 cT2a: 132, cT2b: 91, cT2c: 37 cT3a: 239, cT3b: 61
Gleason score	3 + 3: 22 3 + 4: 93, 4 + 3: 103 4 + 4: 135, 3 + 5: 25 4 + 5: 255, 5 + 4: 34 5 + 5: 3
%PC (%), median (IQR)	41.7 (25.0–60.0)
ADT duration (months), median (IQR)	25.0 (24.0–27.0)
CIRT dose	63 Gy(RBE)/20 fr: 77 57.6Gy(RBE)/16 fr: 298 51.6 Gy(RBE)/12 fr: 295
Early BCR	36 (5.4%)
Time to early BCR (months), median (IQR)	42.8 (29.0–45.3)

ADT: androgen deprivation therapy, BCR: biochemical recurrence, CIRT: carbon-ion radiotherapy, IQR: interquartile range, %PC: percentage of positive biopsy cores, PSA: prostate-specific antigen, SD: standard deviation.

**Table 2:** STAMPEDE risk classification.

Variables	
STAMPEDE	
Risk = 0	58 (8.7%)
Risk = 1	432 (64.5%)
Risk = 2	142 (21.2%)
Risk = 3	38 (5.6%)
STAMPEDE risk factors	
PSA level ≥ 40 ng/mL	78 (11.6%)
cT stage ≥ 3	300 (44.8%)
Gleason score ≥ 8	452 (67.5%)

PSA: prostate-specific antigen, STAMPEDE: Systemic Therapy in Advancing or Metastatic Prostate cancer.

**Table 3:** Clinicopathological characteristics of patients with early BCR and non-early BCR.

Variables	Early BCR (*n* = 36)	Non-Early BCR (*n* = 634)	*p* Value
Age (years), mean (SD)	68.5 (6.3)	68.1 (7.0)	0.69
PSA level (ng/mL), median (IQR)	18.6 (10.8–42.5)	12.5 (7.1–24.1)	0.01
cT stage	cT1: 4 cT2a: 5, cT2b: 5, cT2c: 2 cT3a: 10, cT3b: 10	cT1: 106 cT2a: 127, cT2b: 86, cT2c: 35 cT3a: 229, cT3b: 51	0.03
Gleason score	3 + 4: 4, 4 + 3: 6 4 + 4: 4, 3 + 5: 2	3 + 3: 22 3 + 4: 89, 4 + 3: 97 4 + 4: 131, 3 + 5: 23 5 + 5: 3	0.52
%PC (%), median (IQR)	50.0 (35.1–83.3)	40.0 (25.0–27.0)	<0.01
ADT duration (months), median (IQR)	24.9 (23.7–29.1)	25.0 (24.0–27.0)	0.48
STAMPEDE high-risk (%)	16 (44.4%)	168 (25.9%)	0.02

ADT: androgen deprivation therapy, BCR: biochemical recurrence, IQR: interquartile range, %PC: percentage of positive biopsy cores, PSA: prostate-specific antigen, SD: standard deviation, STAMPEDE: Systemic Therapy in Advancing or Metastatic Prostate cancer.

**Table 4:** Univariate and multivariate analyses of the association between clinical variables and early BCR in high-risk PCa patients after CIRT and ADT.

Variables	Univariate		*p* Value	Multivariate		*p* Value
	Early BCR (%)	Non-Early BCR (%)		HR	95%CI	
Age ≥ 70 vs. < 70 (years)	4.9	5.7	0.77			
PSA ≥ 50 vs. < 50 (ng/mL)	12.5	4.7	0.02	-		0.29
cT3b vs. cT3a-cT1	16.4	4.3	<0.01	3.63	1.76–7.49	<0.01
GS ≥ 9 and 3 + 5 vs. GS ≤ 4 + 4	6.9	4.0	0.07	-		0.41
%PC ≥ 75% vs. < 75%	14.6	3.7	<0.01	3.51	1.84–6.69	<0.01

ADT: androgen deprivation therapy, BCR: biochemical recurrence, IQR: interquartile range, %PC: percentage of positive biopsy cores, PSA: prostate-specific antigen, SD: standard deviation, STAMPEDE: Systemic Therapy in Advancing or Metastatic Prostate cancer.

## Data Availability

The data are contained within the article.
